# An Epidemiological Study of the Quality of Life of Children With Beta-Thalassemia Major (β-TM) and Its Correlates in Kolkata, West Bengal, India

**DOI:** 10.7759/cureus.36888

**Published:** 2023-03-29

**Authors:** Bijit Biswas, Narendra N Naskar, Keya Basu, Aparajita Dasgupta, Rivu Basu, Bobby Paul

**Affiliations:** 1 Community and Family Medicine, All India Institute of Medical Sciences, Deoghar, IND; 2 Public Health Administration, All India Institute of Hygiene and Public Health, Kolkata, IND; 3 Pathology, Calcutta National Medical College and Hospital, Kolkata, IND; 4 Preventive and Social Medicine, All India Institute of Hygiene and Public Health, Kolkata, IND; 5 Community Medicine, Radha Gobinda (RG) Kar Medical College and Hospital, Kolkata, IND

**Keywords:** social discrimination, knowledge, caregivers, quality of life, children, β-thalassemia

## Abstract

Background and objectives

In contrast to their peers who are healthy, children with thalassemia disease are likely to have a lower quality of life (QoL). Knowledge of attributes affecting the QoL of thalassemic children may help identify key areas of intervention to improve it. Thus, the current study was envisioned to find out the quality of life (QoL) of children with beta-thalassemia major (β-TM) and its various correlates.

Methods

Between May 2016 and April 2017, an institution-based cross-sectional observational study was conducted in the thalassemia unit of Calcutta National Medical College and Hospital (CNMC&H), Kolkata, West Bengal, India. During the study period, 328 β-TM children and their carers were interviewed using a structured schedule.

Results

In the final multivariable logistic regression model, thalassemic children who were residing in an urban area (adjusted odds ratio (AOR) (95% confidence interval (CI)): 2.1 (1.1-4.0)), had mothers with a higher educational level (middle and above) (AOR (95%CI): 2.1 (1.1-4.0)), had working parents (AOR (95%CI): 2.7 (1.2-6.3)), had no family history of thalassemia (AOR (95%CI): 3.5 (1.6-8.0)), received less number of blood transfusion in the previous year (<12) (AOR (95%CI): 2.1(1.1-4.2)), had higher pre-transfusional hemoglobin (Hb) level (AOR (95%CI): 1.7(1.1-2.6)), had no transfusion-transmitted infections (TTIs) (AOR (95%CI): 2.8 (1.5-5.2)), had higher body mass index (BMI) Z score (AOR (95%CI): 1.6 (1.1-2.2)), and had higher Carer Quality of Life (CarerQoL) score (>5) (AOR (95%CI): 3.2 (1.6-6.2)) were more likely to have favorable QoL (Pediatric Quality of Life Inventory (PedsQL) score > 54.3).

Interpretation and conclusions

The QoL of the study participants was significantly correlated with their carers’ CarerQoL, mother’s educational level, parent’s working status, place of residence, family history of the disease, blood transfusion frequency, pre-transfusional Hb level, and nutritional and comorbidity status.

## Introduction

Thalassemia is the most common single-gene, autosomal recessive, inherited disease, impacting over 200 million individuals worldwide [1]. It is becoming a global public health concern due to its high incidence in the Indian subcontinent and Southeast Asia, which are recognized hotspots for the condition [2,3]. The thalassemia belt, which runs from the Mediterranean region to the Middle East, the Indian subcontinent, and Southeast Asia, includes India. The country accounts for up to 10% (10,000) of all children born each year with beta-thalassemia major (β-TM). One out of every 10 residents of West Bengal (the fourth most populous state in the nation) was reported to be a thalassemia carrier, a rate that was quite higher than the projected national average of 3%-4% [3-5].

Like any other chronic illness in children, thalassemia can have a number of negative physical and psychological repercussions on a child’s quality of life (QoL). Thalassemia’s effects on a child’s physical development can result in physical deformities, growth retardation, delayed hemolysis, etc. Hepatosplenomegaly, leg ulcers, gallstones, high-output congestive heart failure, and, in the most severe cases, mortality within the first 10 years of life are all consequences of hemolytic anemia. Short stature and a bulged abdomen due to splenomegaly may affect the self-esteem of an affected child. The complications of the disease not only increase the morbidity of the children but also affect their physical, mental, and social functioning, lowering their overall QoL [1,6-8].

Today, advancements in transfusion and iron chelation therapy have extended the life expectancy of β-TM children, but their QoL remains poor compared to their healthy counterparts [9,10]. Before we can intervene, we must first comprehend the various aspects of β-TM children’s QoL. Various sociodemographic (i.e., age, residence, parents’ qualifications, family history of the disease, etc.), socioeconomic (i.e., family income, parents’ employment, etc.), and clinico-therapeutic (i.e., age at diagnosis, blood transfusion frequency, hemoglobin (Hb) level, spleen status, comorbidities, nutritional status, etc.) factors were reported to influence their QoL [11-24]. There may be several other additional attributes, such as the caregiver’s care-related quality of life (CarerQoL), health-seeking behavior (HSB) for their ward, knowledge related to disease, and social discrimination, that are likely to influence the QoL of a thalassemic child. As per our knowledge, only an old Indian study [8] with a relatively small sample size threw some light on the relationship in the QoL of a thalassemic child and his/her carer. Prior studies examined the effect of none of these additional attributes mentioned earlier on the QoL of β-TM children. Thus, to bring about a better understanding of the QoL of β-TM children and its various correlates, the current study was envisioned.

The preprint version of this article was previously posted to the Research Square preprint server on October 21, 2022.

## Materials and methods

The study was an institution-based observational study with a cross-sectional design. It included 328 β-TM children and their caregivers. Our method for data collection was face-to-face interviewing in the thalassemia unit outpatient department (OPD) of Calcutta National Medical College and Hospital (CNMC&H), Kolkata, West Bengal, India, from May 2016 to April 2017 with a structured schedule.

The following elements were included in the schedule: sociodemographic (age, education, residence, and family history of the disease), socioeconomic (working status, per capita monthly income (PCMI)), and clinico-therapeutic (age at diagnosis, blood transfusion frequency, transfusion-transmitted infections (TTIs), last pre-transfusional Hb level, nutritional status, spleen status, and iron chelation status) factors, caregivers’ knowledge of the disease, and perceived level of social discrimination; Pediatric Quality of Life Inventory (PedsQL) [25] and CarerQoL [26] were used for QoL assessment of thalassemic children and their accompanying carers, respectively. Notably, only known β-TM children were enrolled in the study. The disease of these children was diagnosed by the medical officer of the thalassemia unit. The criteria used for diagnosis were fetal hemoglobin (HbF) > 70% with adult hemoglobin (HbA) < 30% on Hb electrophoresis and Hb level < 7 g/dL at the time of diagnosis. These diagnosed β-TM children are evaluated on a regular basis (clinical examination and Hb estimation) by the medical officer of the thalassemia unit for further management (i.e., blood transfusion, vaccination, and iron chelation) to maintain their vitality.

**Figure 1:**
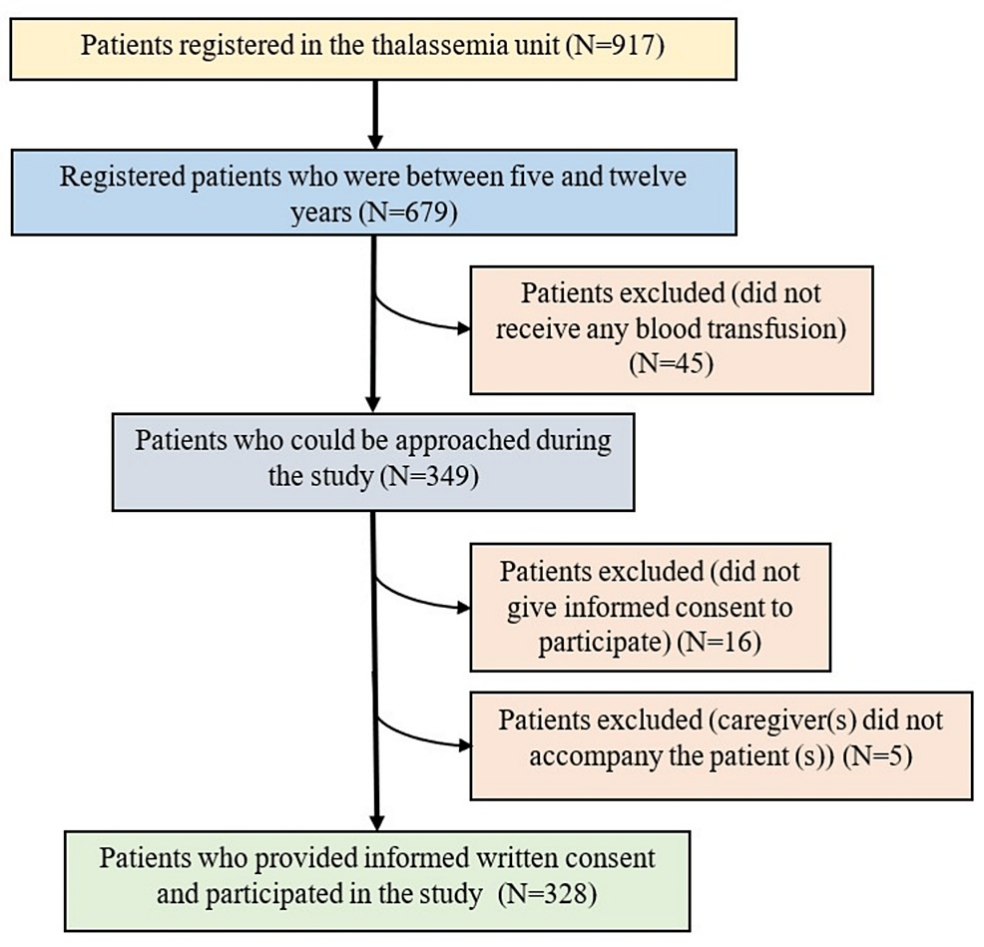
Flowchart showing the selection of study participants (N=328)

Using the formula 4Zα^2^S^2^/d^2^, the minimum sample size for the study is calculated to be 317, using the maximum standard deviation (SD) of QoL score reported by a previous study in West Bengal [13] as 15.3 (in school domain), assuming 5% absolute error, a design effect of 2, and a nonresponse rate of 10%. Here, Zα is the standard normal deviate, S is the standard deviation of the variable, and d is the desired error [27]. Children who were accompanied by a carer and had received at least one blood transfusion last year were approached for the study. Meanwhile, critically ill β-TM children were excluded. Here, critically ill refers to those who needed urgent medical intervention on the day of their visit for survival. The data was collected weekly once. On average, 15-20 β-TM children attend the thalassemia unit’s OPD on a given working day. The principal investigator who was a junior resident doctor then approached every second patient in the OPD and asked them to participate in the study. On average, eight patients and their carers could be interviewed in a single day. In this manner, 328 study participants could be enrolled in the study over 41 data collection days over the course of a year, excluding OPD holidays and per capita interview time of 15-20 minutes. Before beginning each interview, the study participants were asked whether they had been approached before for the study to avoid duplication. In this way, in a year, 328 study participants could be enrolled in the study (Figure 1).

Operational definitions

Some of the operational definitions used in the study were the following: PedsQL score, CarerQoL score, caregiver, caregivers’ disease knowledge, transfusion-transmitted infections, splenomegaly, social discrimination, and health-seeking behavior.

PedsQL Score

Items of the PedsQL scale were reverse-scored. Then, they were linearly transformed to a 0-100 scale, where 0=100, 1=75, 2=50, 3=25, and 4=0. Scores were obtained by summing the items answered. In this way, the total score was calculated, where a higher score indicated a more favorable QoL [25]. The total QoL score was not normally distributed, and the Shapiro-Wilk test was significant. Thus, the QoL score was divided into two outcomes (favorable and unfavorable) by its median (54.3) to perform a logistic regression analysis. In the present study, those who scored more than the median achieved score (54.3) were considered to have favorable QoL.

CarerQoL Score

The scores for all seven items of the scale were added to obtain the total score. The higher the score, the better the care situation. A CarerQoL score of 0 represents the worst care situation, while a score of 7 represents the best care situation [26]. The CarerQoL score was divided into two outcomes (favorable and unfavorable) by its median (5) for analysis.

Caregiver

In the current study, a carer was defined as any adult relative who accompanied the thalassemic child during a visit to the hospital’s thalassemia OPD and is currently living with and caring for the patient.

Caregivers’ Disease Knowledge

This was calculated by adding the scores they received for each knowledge item, with a higher score indicating a higher level of disease knowledge. Those who had knowledge greater than or equal to 4 (median achieved score) were considered to have satisfactory knowledge regarding the disease. Details are described elsewhere [28].

Transfusion-Transmitted Infections (TTIs)

Those who were reported to be positive for hepatitis B surface antigen (HBsAg), anti-hepatitis C virus (HCV), and anti-HIV1/HIV2 as per their medical records were considered to be hepatitis B-, hepatitis C-, and HIV-positive, respectively.

Splenomegaly

It was assessed by palpation of the abdomen of the patient in a supine position and expressed in centimeters.

Social Discrimination

It was assessed by asking the carer, “Do you face any social discrimination for being a carer of a thalassemic child?” Those who reported it to be “yes” were considered to be facing social discrimination.

Health-Seeking Behavior

Those who have continued to consult an allopathic doctor only for their wards’ illness right from its diagnosis were considered to have satisfactory HSB, while others were deemed to have unsatisfactory HSB.

Statistical analysis

Data were analyzed using Statistical Package for the Social Sciences (SPSS) version 16 (SPSS Inc., Chicago, IL, USA). At first, bivariate analysis using the Spearman rho correlation coefficient was done to find out the one-to-one relationship between QoL and its various attributes. Then, the significant variables in the bivariate analysis were entered domain-wise (sociodemographic, clinico-therapeutic, carer’s perception, and CarerQoL) in the hierarchical multivariate logistic regression models using the forced entry method to find out the multivariable correlates of QoL of the study subjects. All the multivariable models were adjusted with PCMI, despite being insignificant in the bivariate analysis, to make the findings of the study more robust. The strength of associations was assessed by odds ratios (OR) at a 95% confidence interval (CI) with a significance level of p<0.05.

Ethical considerations

The Institutional Ethics Committee (IEC) of CNMC&H (approval number: CNMC/7) and All India Institute of Hygiene and Public Health (AIIH&PH), Kolkata, India, cleared the study. Before participating in the study, each carer provided written informed consent, as did the β-TM children. Additionally, the confidentiality of data was assured during collection, analysis, and reporting.

## Results

**Figure 2:**
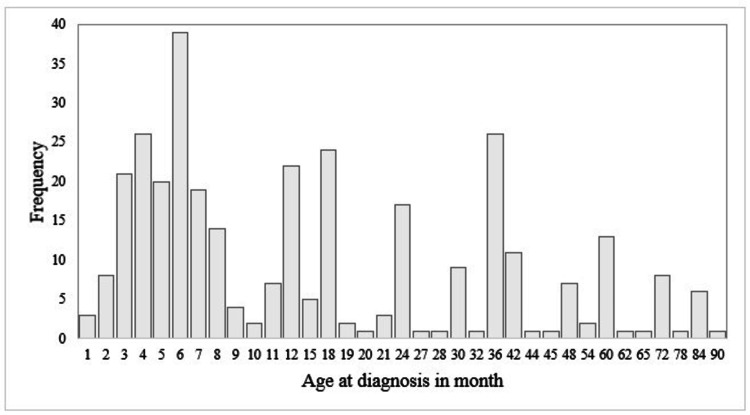
Bar chart showing the distribution of thalassemic children as per their age at the time of diagnosis of thalassemia (N=328)

Most of the β-TM children enrolled in the study belonged to the 11-12 age group (37.2%), with a range of 5-12 years. There was almost equal representation of both sexes. Most of the interviewed carers were parents (98.8%) of the study subjects. The mean ages of mothers and fathers were 29.8 and 34.8 years, respectively. The educational level of most of the parents of the study participants was primary or above (fathers: 58.6%, mothers: 59.8%), whereas more than one-fifth of them were illiterate. The majority of the participants (67.4%) developed symptoms of the disease within the first year of their lives. The median age of the study subjects at the time of presentation of the disease symptoms was six months. More than half (56.5%) of the β-TM children were diagnosed within the first year of their lives, with a median age at diagnosis of 12 months (range: 1-90 months). Three-fifths (61.3%) of them had a palpable spleen. The palpable spleen size ranged from 1 to 8 cm. The majority of them (63.7%) received blood transfusions once or less than once a month. Most of them (35.4%) had a pre-transfusional Hb level between 5.3 and 5.9 g/dL. Two-fifths of them (39.9%) were suffering from TTIs, of which 34.5% were anti-HCV-positive, while 4.3% and 1.8% were HBsAg- and anti-HIV-positive, respectively (Table 1 and Figure 2).

**Table 1:** Background characteristics of the study participants (N=328) SD: standard deviation, PCMI: per capita monthly income, USD: United States dollar

Variable	Number (%)/mean ± SD
Age of the patient in completed years	8.0 ± 2.3
Sex of the patient	
Male	177 (54)
Female	151 (46)
Caregiver interviewed (concerning the patient)	
Father	78 (23.8)
Mother	246 (75)
Grandmother	3 (0.9)
Grandfather	1 (0.3)
Age of the caregiver in completed years	32.0 ± 6.7
Sex of the caregiver	
Male	79 (24.1)
Female	249 (75.9)
Educational level of the father (completed years of schooling)	
Illiterate (0)	70 (21.3)
Below primary (0-4)	66 (20.1)
Primary (5-7)	76 (23.2)
Middle (8-9)	74 (22.6)
Secondary and above (≥10)	42 (12.8)
Educational level of the mother (completed years of schooling)	
Illiterate (0)	71 (21.6)
Below primary (0-4)	61 (18.6)
Primary (5-7)	84 (25.6)
Middle (8-9)	68 (20.7)
Secondary and above (≥10)	44 (13.5)
Both parents work for pay (yes)	62 (18.9)
PCMI in USD	23.3 ± 12.5
Place of residence	
Urban	91 (27.7)
Rural	237 (72.3)
Family history of thalassemia (yes)	67 (20.4)
Number of blood transfusion received by the patient in the previous year	11.6 ± 4.9
Last pre-transfusional hemoglobin level of the patient in g/dL	5.5 ± 0.8
Transfusion-transmitted infections (yes)	131 (39.9)
Whether the patient had undergone splenectomy (yes)	83 (25.3)
Size of the palpable spleen of the patient in cm	3.9 ± 2.6
Whether the patient is on iron chelators (yes)	306 (93.3)
Duration since the patient receives iron chelators in months	35.6 ± 20.2
Caregiver knowledge score regarding the disease	3.4 ± 1.5
Caregiver who had faced social discrimination for being a caregiver of a thalassemic child (yes)	137 (41.8)
Health-seeking behavior (satisfactory)	200 (61)

**Figure 3:**
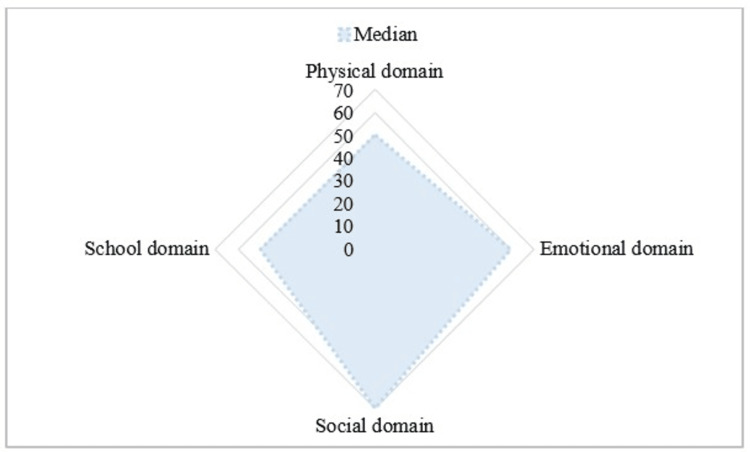
Radar chart showing the contribution of various domains of QoL to the total PedsQL score of the patient (N=328) QoL: quality of life, PedsQL: Pediatric Quality of Life Inventory

The median total PedsQL score was 54.3 with an interquartile range (IQR) of 43.5-67.4, while the median CarerQoL score was 5 with an IQR of 4-7. Considering individual domains of PedsQL, the school domain was most affected, followed by physical, emotional, and social domains (Figure 3). The bivariate strength of the association between the QoL of the study participants and its various correlates is depicted in Table 2.

**Table 2:** Spearman rho correlation matrix showing various correlates of the favorable quality of life of the study subjects (N=328) A: age of the patient (increasing), B: sex of the patient (female), C: fathers’ educational level (middle and above), D: mothers’ educational level (middle and above), E: both parents work for pay (yes), F: PCMI (increasing), G: place of residence (urban), H: family history of thalassemia (no), I: blood transfusion frequency (<12 times), J: last pre-transfusional Hb level (increasing), K: TTIs (no), L: patients’ BMI Z score (increasing), M: undergone splenectomy (yes), N: the patient is on iron chelators (yes), O: caregivers’ knowledge level (satisfactory: ≥4), P: caregiver faced discrimination (no), Q: health-seeking behavior (satisfactory), R: CarerQoL score (favorable: >5), S: PedsQL score (favorable: >54) **Correlation was significant at the 0.01 level. *Correlation was significant at the 0.05 level. PCMI: per capita monthly family income, Hb: hemoglobin, TTIs: transfusion-transmitted infections, BMI: body mass index

	A	B	C	D	E	F	G	H	I	J	K	L	M	N	O	P	Q	R	S
A	1.0	0.0	0.1^**^	0.1	0.2^**^	0.1^*^	-0.1^*^	0.0	-0.2^**^	0.2^**^	0.0	0.3^**^	0.3^**^	0.0	0.1	0.1	0.0	0.1^*^	0.1^*^
B		1.0	-0.1^*^	-0.1	0.0	0.0	-0.1	-0.1^*^	-0.1	0.1	0.0	-0.1	0.1	0.0	0.1	0.0	0.1	0.0	0.1
C			1.0	-0.3^**^	0.0	0.2^**^	0.0	0.3^**^	0.0	0.0	0.0	0.0	-0.1	0.0	0.0	-0.1^*^	0.0	0.0	0.1
D				1.0	-0.1^*^	0.0	0.0	0.1	0.0	0.0	0.0	0.0	-0.1^*^	0.0	0.1^**^	0.0	0.0	0.1^*^	0.1^*^
E					1.0	0.2^**^	-0.1	-0.1^*^	-0.1^*^	0.2^**^	-0.1	0.1^*^	0.0	0.0	0.0	-0.1	0.0	0.1^*^	0.2^**^
F						1.0	-0.1	-0.1	0.0	0.1	0.0	0.0	0.0	-0.1	0.1	0.0	0.2^**^	0.0	0.0
G							1.0	-0.2^**^	-0.2^**^	0.0	0.1^*^	0.0	0.0	0.1^*^	0.2^**^	0.2^**^	0.2^**^	0.1^*^	0.3^**^
H								1.0	0.1^**^	0.1	0.0	0.1	0.1	0.0	0.0	0.1	0.2^**^	0.1	0.3^**^
I									1.0	0.4^**^	-0.1	0.4^**^	0.3^**^	0.1^*^	0.1	-0.1	0.1	0.2^**^	0.4^**^
J										1.0	0.0	0.2^**^	0.3^**^	-0.1	0.1	-0.1	0.0	0.1	0.3^**^
K											1.0	-0.1	0.0	0.0	-0.1^*^	0.2^**^	-0.1	0.2^**^	0.3^**^
L												1.0	0.0	0.0	0.1	0.0	0.1	0.2^**^	0.3^**^
M													1.0	0.0	0.1	0.1^*^	0.0	-0.1^*^	0.1^*^
N														1.0	0.0	0.0	0.0	-0.1	0.0
O															1.0	-0.2^**^	0.4^**^	0.2^**^	0.3^**^
P																1.0	0.1^*^	0.3^**^	0.3^**^
Q																	1.0	0.2^**^	0.3^**^
R																			0.4^**^
S																			1.0

**Figure 4:**
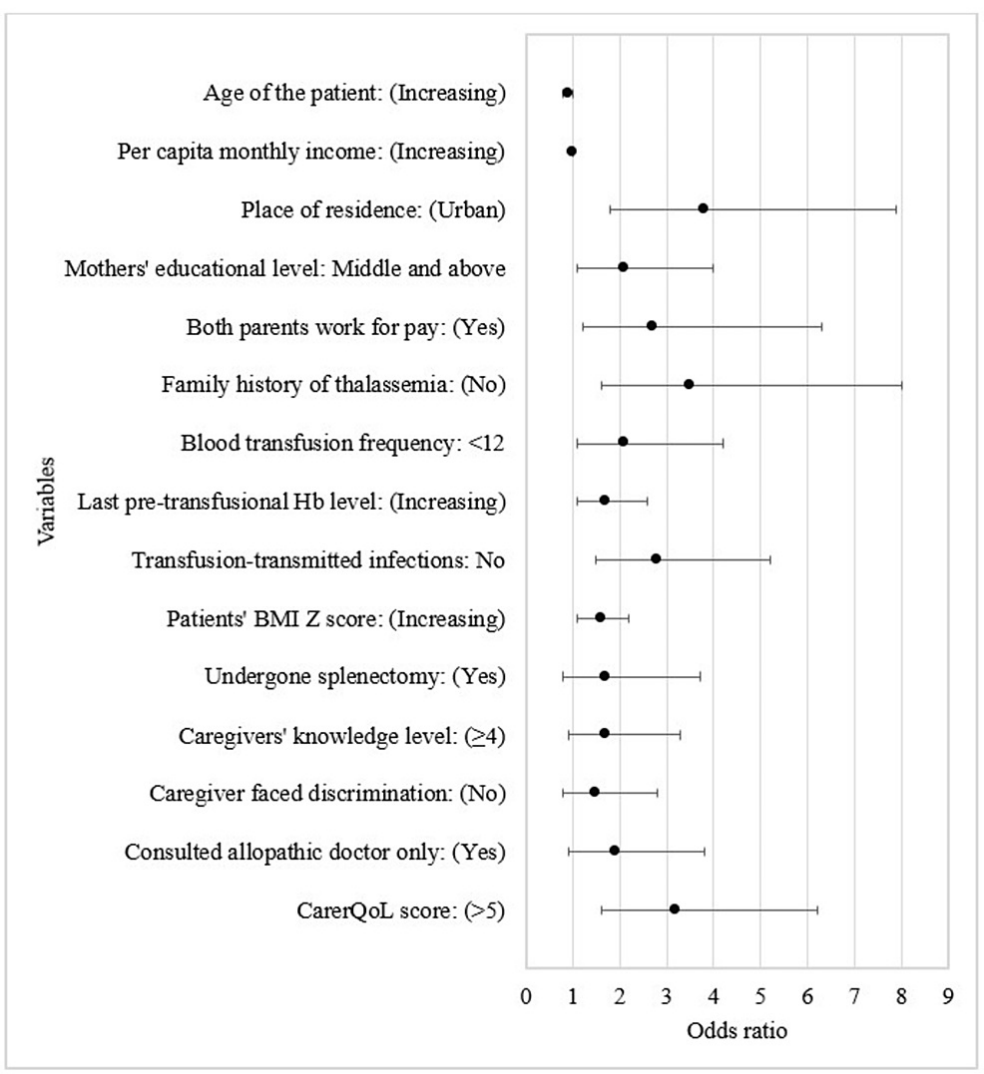
Forest plot showing predictors of the quality of life of thalassemic children (N=328) Hb: hemoglobin, BMI: body mass index, CarerQoL: Carer Quality of Life

In the final multivariable logistic regression model, thalassemic children who lived in an urban area (adjusted odds ratio (AOR) (95%CI): 3.8 (1.8-7.9)), had mothers with a higher educational level (middle and above) (AOR (95%CI): 2.1 (1.1-4.0)), had working parents (AOR (95%CI): 2.7 (1.2-6.3)), had no family history of thalassemia (AOR (95%CI): 3.5 (1.6-8.0)), received fewer blood transfusions (<12) (AOR (95%CI): 2.1(1.1-4.2)), had higher pre-transfusional Hb level (AOR (95%CI): 1.7 (1.1-2.6)), had no TTIs (AOR: 2.8 (1.5-5.2)), had higher body mass index (BMI) Z score (AOR (95%CI): 1.6 (1.1-2.2)), and had higher carers’ CarerQoL score (>5) (AOR (95%CI): 3.2 (1.6-6.2)) were more likely to have favorable QoL (PedsQL score > 54.3). The multivariable model was adjusted with their age, PCMI, spleen status, HSB, carer’s knowledge level related to the disease, and social discrimination status. Overall, the model explained 55.1% of the variability of the QoL of the study participants with a predictive accuracy rate (PAR) of 82.9%, while their carers’ CarerQoL score independently explained 17.9% of its variability with a PAR of 68.3% (Table 3 and Figure 4).

**Table 3:** Hierarchical multivariable logistic regression analysis showing predictors of favorable quality of life of thalassemic children (N=328) SD: standard deviation, PedsQL: Pediatric Quality of Life Inventory, CarerQoL: Carer Quality of Life, AOR: adjusted odds ratio, CI: confidence interval, PCMI: per capita monthly income, USD: United States dollar, BMI: body mass index, TTIs: transfusion-transmitted infections, QoL: quality of life, Hb: hemoglobin

Variables	Favorable quality of life (PedsQL > 54.3 (median))				
	Mean (SD)/number (%)	Model 1	Model 2	Model 3	Model 4
		AOR (95%CI)	AOR (95%CI)	AOR (95%CI)	AOR (95%CI)
Sociodemographic domain					
Age of the patient (increasing)	8.3 (2.3)	1.1 (0.9-1.2)	0.9 (0.8-1.1)	0.9 (0.8-1.1)	0.9 (0.8-1.0)
PCMIin USD (increasing)	23.5 (12.8)	1.0 (1.0-1.0)	1.0 (1.0-1.0)	1.0 (0.9-1.0)	1.0 (1.0-1.0)
Place of residence					
Urban	70 (76.9)	4.1 (2.3-7.3)	4.4 (2.2-8.6)	3.4 (1.7-7.0)	3.8 (1.8-7.9)
Rural	98 (41.4)	Reference	Reference	Reference	Reference
Mothers’ educational level					
Middle and above	67 (59.8)	1.9 (1.1-3.2)	2.3 (1.3-4.1)	2.2 (1.2-4.2)	2.1 (1.1-4.0)
Below middle	101 (46.8)	Reference	Reference	Reference	Reference
Both parents work for pay					
Yes	45 (72.6)	2.9 (1.5-5.6)	2.7 (1.3-5.9)	2.9 (1.3-6.7)	2.7 (1.2-6.3)
No	123 (46.2)	Reference	Reference	Reference	Reference
Family history of thalassemia					
No	152 (58.2)	3.5 (1.8-6.9)	3.5 (1.7-7.5)	3.4 (1.6-7.5)	3.5 (1.6-8.0)
Yes	16 (23.9)	Reference	Reference	Reference	Reference
Patient’s clinico-therapeutic domain					
Blood transfusion frequency					
<12 units	112 (70)		2.7 (1.5-5.0)	2.7 (1.4-5.2)	2.1 (1.1-4.2)
≥12 units	56 (33.3)		Reference	Reference	Reference
Last pre-transfusional Hb level in g/dL (increasing)	5.7 (0.8)		1.6 (1.1-2.3)	1.7 (1.1-2.5)	1.7 (1.1-2.6)
TTIs					
No	124 (62.9)		3.7 (2.1-6.5)	3.3 (1.8-6.0)	2.8 (1.5-5.2)
Yes	44 (33.6)		Reference	Reference	Reference
Patients’ BMI Z score (increasing)	0.3 (0.9)		1.5 (1.1-2.1)	1.6 (1.1-2.2)	1.6 (1.1-2.2)
Undergone splenectomy					
Yes	51 (61.4)		1.2 (0.6-2.4)	1.2 (0.6-2.5)	1.7 (0.8-3.7)
No	117 (47.8)		Reference	Reference	Reference
Caregivers’ perception domain					
Caregivers’ knowledge level					
Satisfactory (≥4)	112 (64.7)			1.9 (1.0-3.7)	1.7 (0.9-3.3)
Unsatisfactory (<4)	56 (36.1)			Reference	Reference
Caregiver faced discrimination					
No	118 (61.8)			1.8 (1.0-3.4)	1.5 (0.8-2.8)
Yes	50 (36.5)			Reference	Reference
Health-seeking behavior					
Satisfactory	123 (61.5)			2.1 (1.1-4.1)	1.9 (0.9-3.8)
Unsatisfactory	45 (35.2)			Reference	Reference
Caregivers’ QoL domain					
CarerQoL score					
Favorable (>5)	101 (73.2)				3.2 (1.6-6.2)
Unfavorable (≤5)	67 (35.3)				Reference
NagelkerkeR^2^	-	0.262	0.466	0.523	0.551
R^2 ^change	-	0.262	0.204	0.057	0.028
Omnibus test (p-value)	-	<0.001	<0.001	<0.001	0.001
Hosmer-Lemeshow test (p-value)	-	0.800	0.439	0.093	0.212

## Discussion

This was a cross-sectional, institution-based, observational study aimed at assessing the QoL of children with β-TM and its various correlates.

QoL of the study subjects

We found that the school domain of QoL was most affected, followed by physical, emotional, and social domains. This was in accordance with the findings of a study in West Bengal [13]. Considering the mean QoL score of the present study (54.4±16.0), it was lower compared to all the previous Indian studies [10-14], which is quite bothersome and requires urgent attention and prompt intervention. The reasons for the differences may be the variability in the selection criteria used, age composition, socioeconomic status, clinical profile of the study subjects, sample size, etc. (Table 4).

**Table 4:** QoL scores of thalassemic children as reported by previous Indian studies and the present study SD: standard deviation, QoL: quality of life

Studies		QoL score (mean ± SD)				
	Year	Physical	Emotional	Social	School	Total
Chordiya et al. [11]	2018	78.4±10.4	79.7±13.5	79.8±9.6	60.6±17.2	76.1±14.1
Sharma et al. [10]	2017	88.2±19.5	79.3±17.5	98.1±7.5	75.3±13.5	83.7±10.8
Dhirar et al. [12]	2016	81.3±21.3	75.4±17.4	92.0±17.4	77.8±14.5	82.0±14.4
Saha et al. [13]	2015	57.2±12.0	67.8±11.2	74.4±11.8	49.4±15.3	63.0±9.0
Gupta et al. [14]	2015	84.9±19.6	73.9±15.4	76.3±6.4	67.6±27.4	75.2±2.7
Present study	2020	50.1±20.3	58.5±14.9	62.8±24.2	48.9±31.1	54.4±16.0

Sociodemographic predictors of QoL

In the present study, higher age improved the chances of having a favorable QoL. It was consistent with the findings of the previous studies [11,14,15], but not with the findings of the Delhi study [12]. The difference in findings may be due to study population variation. One probable explanation is that as the child grows older, he or she copes with the disease’s burden. On the other hand, it might also be due to survivorship bias. Considering the place of residence, urban residence emerged as a significant correlate of favorable QoL. An Egyptian study [16] also reported similar observations, while a study in the Gaza Strip [17] failed to demonstrate this association. It may be because rural settings themselves present many challenges, such as long-distance travel to seek healthcare for the disease, which might harm QoL.

We found that the mother’s educational level was one of the significant attributes of favorable QoL, which was in line with the existing literature [16,18]. The reasons could be that an educated mother who is likely to possess more knowledge regarding her child’s ailment renders a better quality of care to her ward, which could have positively influenced their ward’s QoL. We observed that PCMI did not affect the QoL of a thalassemic child, which was similar to a prior study [17]. Those with a family history of thalassemia had a lower QoL in the current study. This finding was concurrent with the results of an Indian study [13]. This may be because those who had a family history of the disease tend to have a fatal form of the disease. Disease severity is a known influencer of QoL.

Clinico-therapeutic predictors of QoL

In the present study, study subjects who had less blood transfusion frequency had more favorable QoL. It was concurrent with the findings of prior studies [11,13,16,18]. This may be because the blood transfusion requirement is directly proportional to the severity of the disease in the case of a thalassemic, which is a known determinant of QoL. On the other hand, splenectomy may be another possible explanation for lower blood transfusion requirements in children with better QoL than others, as we found a significant association between splenectomy and QoL. Further, splenectomy also leads to a reduction in iron overload-related complications and hospital visits in thalassemia. It was in line with the findings of a Sri Lankan study [19]. Similarly, we discovered that the last pre-transfusional Hb level was an important associate of QoL. This was similar to the findings of an Indian study [13] and several foreign studies [15,20,21], which reported similar observations. It may be so because the optimal Hb level in the blood at any given point of time directly influences the physical and mental functioning and the overall QoL of a thalassemic child, just as it does for any other human being.

We found that TTI had a negative impact on the subjects’ QoL. This was in line with the existing evidence [12,18,22,23], which reported the negative impact of comorbidity on the QoL of a thalassemic. This may be because the additional burden of TTI over thalassemia significantly affects the QoL of an affected child. Considering nutritional status, we found that a higher BMI Z score predicted favorable QoL. This observation was supported by the findings of a Sri Lankan [19] and an Egyptian [24] study. It may be because a well-nourished child is more empowered to cope up with a chronic disease like thalassemia. Considering iron chelation, an Indian study [12] reported that those who were not on any form of iron chelators had a favorable QoL, which we did not find. Some foreign studies [18,24] also reported an association between serum ferritin level and QoL, which we did not examine due to the non-documentation of that data in the present study setting.

Caregivers’ perception-related predictors of QoL

In the present study, caregivers’ knowledge regarding the disease significantly influenced their wards’ QoL. The reasons could be attributed to better HSB (timely relevant management of the child’s ailment) and higher resilience (to cope with disease burden) in knowledgeable carers compared to others. Maybe that is why we found HSB to be an important attribute of QoL in our study. It is already known that thalassemia does not have any proven non-allopathic management. Seeking treatment from a non-allopathic physician may thus provide a false sense of security, delaying the appropriate management of an affected child to maintain his or her vitality. It endangers both their QoL and life. Similarly, we found that the carer’s social discrimination status had a negative impact on the QoL of his or her ward. It may be because a socially discriminated carer may curse their ward for the ailment owing to the mental disturbance caused by the faced discrimination. This could have a negative impact on a child’s self-esteem and QoL. Some common discriminating behaviors a carer of a thalassemic child usually faces are blame and avoidance from relatives and even strangers, as reported by studies in Iran [29] and the USA [30].

Influence of caregivers’ QoL on patients’ QoL

We found that the QoL of the accompanying carer significantly influences the QoL of a thalassemic child. This was in concordance with the findings of a prior Indian study [8]. This may be because a carer with a better CarerQoL renders a better quality of care to his/her ward, which positively influences their QoL.

Contribution of the current study to the existing literature

This study confirms age, place of residence, the mother’s educational level, PCMI, blood transfusion frequency, pre-transfusional Hb level, TTIs, and spleen status as significant influencers of the QoL of a thalassemic child. Additionally, it enriched the existing literature by establishing the carer’s social discrimination status, knowledge related to the disease, HSB for his/her ward, and CarerQoL as significant attributes of QoL of thalassemic children.

Limitations of the study

Firstly, as the study was cross-sectionally designed, a causal association between QoL and its various attributes could not be ascertained. Secondly, for most of the data, we relied upon the caregiver’s self-reporting. Thus, there may be response- and social desirability-related biases that might have influenced our results. Thirdly, in the present study, CarerQoL and the knowledge of the accompanying carers, who may not be the principal carer (whose CarerQoL and knowledge might be more relevant) in every instance, related to the disease were assessed. Fourthly, as it was an institution-based study, the effect of environmental conditions (i.e., housing type, overcrowding, etc.) on the QoL of the study subjects could not be assessed, which are known determinants of QoL. Finally, we could not assess the effect of serum ferritin levels on the QoL of the study subjects due to the non-documentation of that data in the present study setting.

## Conclusions

The QoL of the study participants was significantly correlated with their carers’ CarerQoL, mother’s educational level, parent’s working status, place of residence, family history of the disease, blood transfusion frequency, pre-transfusional Hb level, and nutritional and comorbidity status. Among these CarerQoL of the carers, blood transfusion frequency, pre-transfusional Hb level, and nutritional status were the modifiable attributes. Thus, these modifiable attributes must be prioritized in interventions targeted at improving the QoL of thalassemic children. Doctors treating these children must abide by existing guidelines on disease management to improve their clinico-therapeutic profile. Nutritional support must be provided for every thalassemic child. Caregivers of these children should be socially, psychologically, and, if possible, financially supported. On every given opportunity, awareness related to the disease must be raised in the affected families and communities to improve their HSB, alleviate the stigma related to the disease, and avert the birth of a thalassemic.
